# Neutrophil responses are substantially prolonged and robust following early postnatal chorda tympani or lingual nerve transection

**DOI:** 10.1007/s00221-026-07303-z

**Published:** 2026-04-27

**Authors:** Jacquelyn M. Omelian, Andrew J. Riquier, Suzanne I. Sollars

**Affiliations:** 1 Department of Psychology, University of Nebraska at Omaha, 6001 Dodge Street, 419 Allwine Hall, Omaha, NE 68182 USA; 2 Department of Psychology, University of Nebraska-Lincoln, Lincoln, USA 1220 T St 224 Burnett Hall, NE 68588

**Keywords:** Chorda tympani, Lingual nerve, Trigeminal, Myeloperoxidase, Development

## Abstract

While the mature gustatory and trigeminal systems are capable of regeneration following surgical nerve transection, transections occurring early in life result in permanent effects on innervated tissues. Converging evidence suggests that facilitators of the innate immune response, such as neutrophils, may contribute to these developmental differences in injury recovery. In the current study, Sprague-Dawley rats underwent transection of the trigeminal lingual nerve (LX) or gustatory chorda tympani nerve (CTX) at either 10, 25, or 65 days of age. Tongues were extracted 12, 24, or 48 h post-surgery, and MPO+ leukocyte (neutrophil) densities in the taste buds and surrounding muscle and epithelium were assessed. The neutrophil response to LX or CTX performed at 25 or 65 days of age was minimal to non-existent at all time points examined. In contrast, we observed a robust neutrophil response on both the ipsilateral and contralateral sides of the tongue starting 12 h after LX or CTX performed at 10 days of age, continuing through 24 h post-surgery, before returning to baseline quantities at 48 h. These results add to growing evidence that differences in the innate immune response may contribute to developmental differences in gustatory and trigeminal system injury recovery.

## Introduction

On the anterior tongue, fungiform taste buds are innervated by the gustatory chorda tympani nerve (CT) while the perigemmal tissue is innervated by the somatosensory lingual nerve (LN). These spatially adjacent tissue types provide a unique model for developmental differences in neuroplasticity. In adult rats (> 40 days postnatal; P40) a surgically transected LN (LX) or CT (CTX) regenerates in weeks, with minimal long-term consequences (Sollars 2005; Omelian et al. 2016). However, identical surgeries performed at ≤ P10 (neonatal) result in limited (LX; Omelian et al. 2016) and non-existent regeneration (CTX; Sollars et al. 2002; Sollars 2005). While the precise reasons for this developmental difference in recovery are unclear, the innate immune system is known to contribute variably to injury recovery outcomes (Nadeau et al. 2011; Kurimoto et al. 2013). Interleukin-1 (IL-1) is vital to taste function recovery and necessary for regeneration following CTX in adult mice (Shi et al. 2012; Dong et al. 2023). Polymorphonuclear leukocytes, or neutrophils, are short-lived (18–24 h life span; Wright et al. 2010; Kubes 2018), hematopoietic stem cell-derived innate immune phagocytes that rapidly infiltrate damaged or infected tissue, typically peaking and diminishing within 24–48 h (Levine et al. 1990; Kolaczkowska and Kubes 2013; Selders et al. 2017). Neutrophils are necessary for sciatic and optic nerve injury recovery (Nadeau et al. 2011; Kurimoto et al. 2013) and their influence on CT regeneration appears to be time and magnitude sensitive. Neutrophils increase in number in the tongue following adult CTX prior to regeneration, yet prolonging this response is deleterious to recovery (Steen et al. 2010). Further, aged rats have a 300% larger neutrophil response to CTX and have substantially less recovery than younger adults (He et al. 2012). However, neutrophil assessments have not yet been conducted following neonatal CTX, where recovery does not occur at all, nor following LX, where recovery ranges from partial to complete based on age. In the current study, we assessed the timing, location, and magnitude of the neutrophil response in the tongue following LX or CTX performed in neonatal, juvenile, and adult rats.

## Materials and methods

### Subjects

The current study used female Sprague-Dawley rats (*n* = 117) bred at the University of Nebraska at Omaha. Animals were housed in Plexiglas cages with corncob bedding, with *ad libitum* access to food and water in temperature- and humidity-controlled rooms on a 12/12 light/dark cycle. Litters were kept to 6–12 pups/dam and pups were weaned at 25 days of age (day of birth being P0). Based on representative time points of rat gustatory development (Sollars 2005), rats underwent surgery (CTX, LX, or sham) at either 10 (neonate), 25 (juvenile) or 65 (adult) days of age. All experimentation involving live animal subjects was carried out with the approval of the Institutional Animal Care and Use Committee of the University of Nebraska and all institutional and NIH guidelines were followed.

### Surgeries

Using well-established procedures (Sollars 2005; Omelian et al. 2016; Riquier and Sollars 2017, 2020), 54 rats (*n* = 9/condition) were anesthetized with methohexital sodium and a small incision was made in the medial neck. Unilateral transections were performed on the right side with the left side remaining intact. For CTX, the nerve was sectioned between the anterior belly of the digastricus and the masseter muscles where the CT bifurcates from the lingual branch of the trigeminal nerve. For LX, the digastricus and masseter muscles were bypassed, and the LN was visualized and cut proximal to its juncture with the CT while keeping the CT intact. Age and time-matched sham surgeries (*n* = 54; 9/condition) were completed using identical procedures for each transection model, but the relevant nerve was left intact after it was exposed. To independently evaluate any potential effect of surgery, tissue from non-surgical age-matched control animals was used for comparison. Tissue in the non-surgical rats was collected following animal sacrifice at P11, P26 or P66 (*n* = 3/condition), ages consistent with the 24-hour post-surgical time point.

### Tissue collection and sectioning

For each surgical age (10, 25, or 65 days), 18 rats (9 CTX, 9 LX) were euthanized either 12, 24, 48 h post-surgery (*n* = 3/condition). These time points coincide with the previously reported neutrophil response in adult CTX animals, which peaked and then normalize by 2 days post-surgery (Steen et al. 2010). Tongues were removed, briefly rinsed with double-distilled water, dried, embedded in OCT, flash frozen in methylbutane and stored at -80˚C until sectioning. Beginning at the anterior-most tip, each tongue was sectioned at 10 μm and thaw-mounted on gel coated slides for a total of 150 sequential sections. Several sections of spleen were collected and processed alongside tongue tissue as a positive control.

### Immunohistochemical processing

**Fig. 1:**
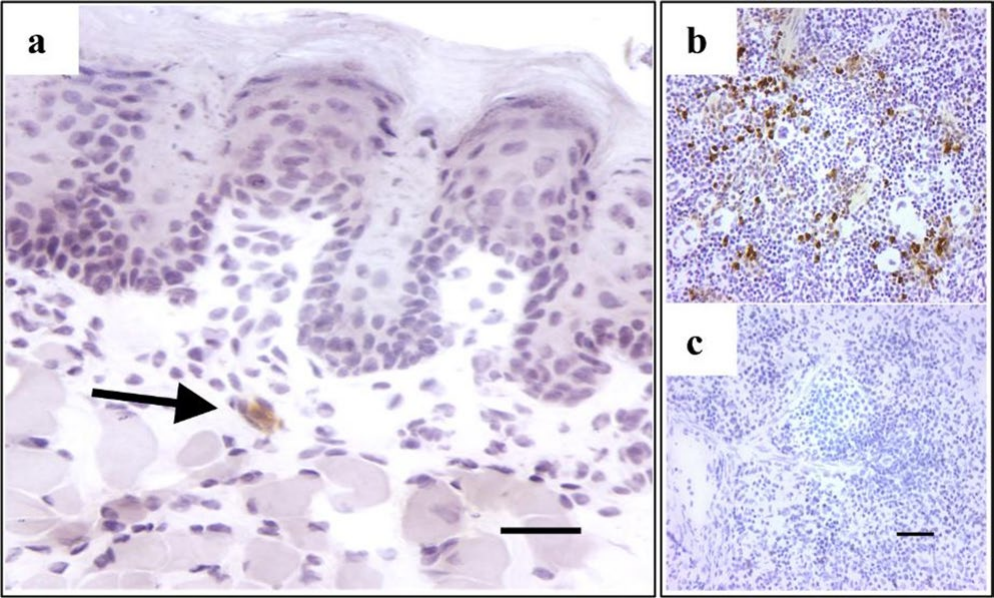
Example neutrophil in the anterior tongue (arrow). MPO+ stained neutrophils appear brown against hematoxylin counterstained tongue tissue (**a**). Examples of positive (**b**) and negative (**c**) control spleen tissue. Scale bars = 50 μm

The presence of neutrophils was assessed using a standardized immunohistochemical staining protocol (Wall and McCluskey 2008; Steen et al. 2010; He et al. 2012). Slide mounted tissue sections were fixed in 0.2% glutaraldehyde in phosphate-buffer solution, followed by room temperature incubation in rabbit anti-rat myeloperoxidase antibody (MPO; 1:200; ThermoFisher). After 48 h, sections were incubated in biotinylated goat anti-rabbit IgG (1:200; Vector Labs), then 2% avidin-biotin complex solution containing 0.1% Triton X-100 (Vector Laboratories) and 5’5 diaminobenzidine (0.05% with 0.01% H2O2; Sigma-Aldrich). Processing was followed by a hematoxylin counterstain and coverslipping with DPX (see Fig. 1). In each immunohistochemistry run, spleen sections with and without the primary antibody were utilized as positive and negative controls, respectively (Fig. 1).

### Microscopy

**Fig. 2:**
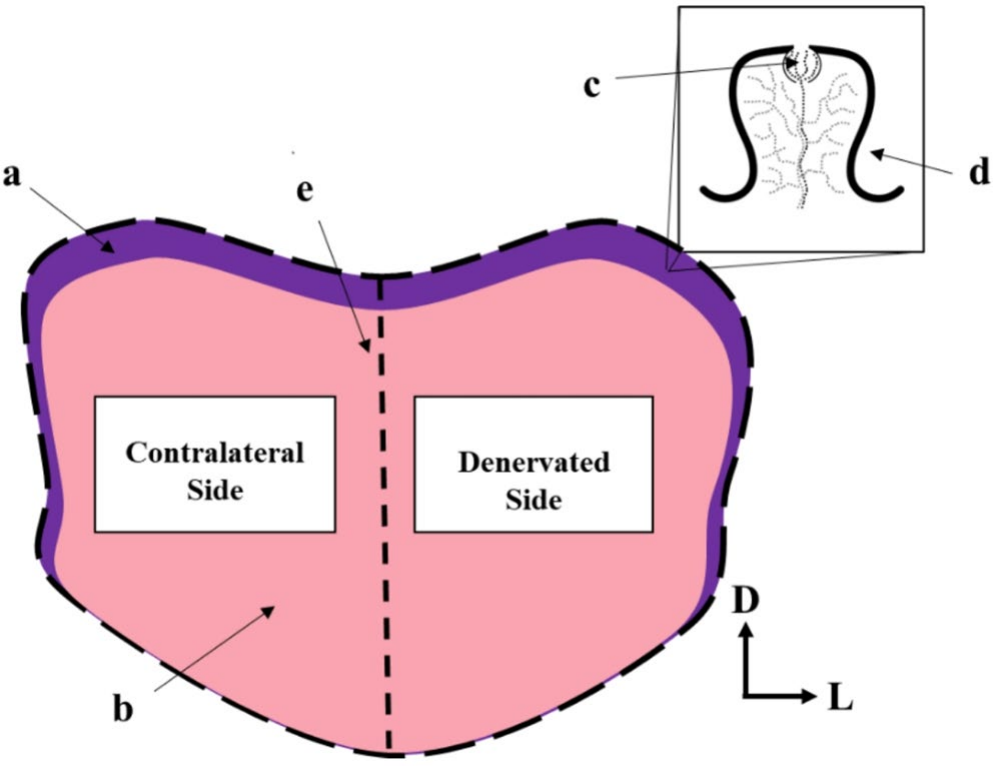
Illustration of quantification measures within a tongue section. (**a**) Epithelium (**b**) Muscle (**c**) Fungiform taste bud (**d**) Fungiform papillae. Dashed lines represent perimeter tracing and midline division (**e**) used to differentiate cut and contralateral sides. Directional arrows indicate (D) dorsal and (L) lateral orientation

Using a Leica microscope and Neurolucida software (MBF Bioscience), cell counts were collected from 10 tongue sections per side per animal, with 50–100 μm between sections ensuring no single neutrophil was double-counted across multiple sections. All counts were performed by a single trained observer while blind to experimental condition. The perimeter of each tongue section was traced, and a vertical division was drawn along the midline to delineate right and left sides of the tongue (Fig. 2). This allowed for comparison of denervated and contralateral tissue in CTX and LX animals, and data collection from a standardized portion of the tongue in sham and non-surgical animals. Each experimental area was systemically analyzed at 20x (X 1.6) for the presence of immunopositive neutrophils. The relative location of the neutrophils in the tongue (fungiform taste bud, fungiform papillae, the epithelial layer, or within the submucosal muscle fibers; See Fig. 2) was recorded and the percent of total neutrophils was calculated for each area.

### Data analysis

MPO+ cells across the 10 sections per animal were summed to create a total neutrophil count for each side (ipsilateral and contralateral to surgery) of the tongue for each animal. To allow for comparisons across age as the tongue grows significantly between neonatal and adult ages (Iwasaki et al. 1997; Hendricks et al. 2004), neutrophil densities were calculated and used for all analyses. Total neutrophil counts were divided by the total volume of the tongue sections in which the cells were counted and multiplied by 100,000. All analyses were performed using SPSS version 31 and an alpha level of 0.05.

Since neutrophils infiltrate both ipsilateral and contralateral sides of the tongue following adult rat CTX (Philips and Hill 1996; Steen et al. 2010; He et al. 2012), age-matched intact, non-surgical animals were used for control comparisons. To ensure any observed neutrophil increases were transection-specific, tongue neutrophil densities on the ipsilateral and contralateral sides of sham animals were also compared to those of intact animals. The average neutrophils density did not significantly differ between sham and intact animals at any time (all *p*’s > 0.1), so sham and intact animals were combined as a single control group for analyses. A one-way ANOVA was utilized to determine if neutrophil density or location differed across development in intact animals aged P10, P25, or P65.

A between-subjects ANOVA was conducted to determine the effects of surgery type (CTX, LX, CTX contralateral, LX contralateral, control), surgical age, and time-point post-surgery, on neutrophil density. Bonferroni post-hoc tests were then performed to generate overall standard errors for the model, which were then used alongside the specific group means to perform planned comparisons for conditions of interest.

## Results

### Tongue neutrophil density and spatial distribution are consistent across development

In order to determine if neutrophil density or location on the tongue differed across development, a one-way ANOVA was performed on intact animals aged 10, 25, or 65 days of age (*n* = 6/age). Neither density (*p* > .1) nor location (*ps* > 0.1) differed as a function of age. The vast majority of neutrophils (> 98%) were present in the muscle, with the remaining located in the epithelium (1%) and papillae (0.69%). No neutrophils were present in the taste buds.

### Developmental differences in the neutrophil response to nerve transection

**Fig. 3:**
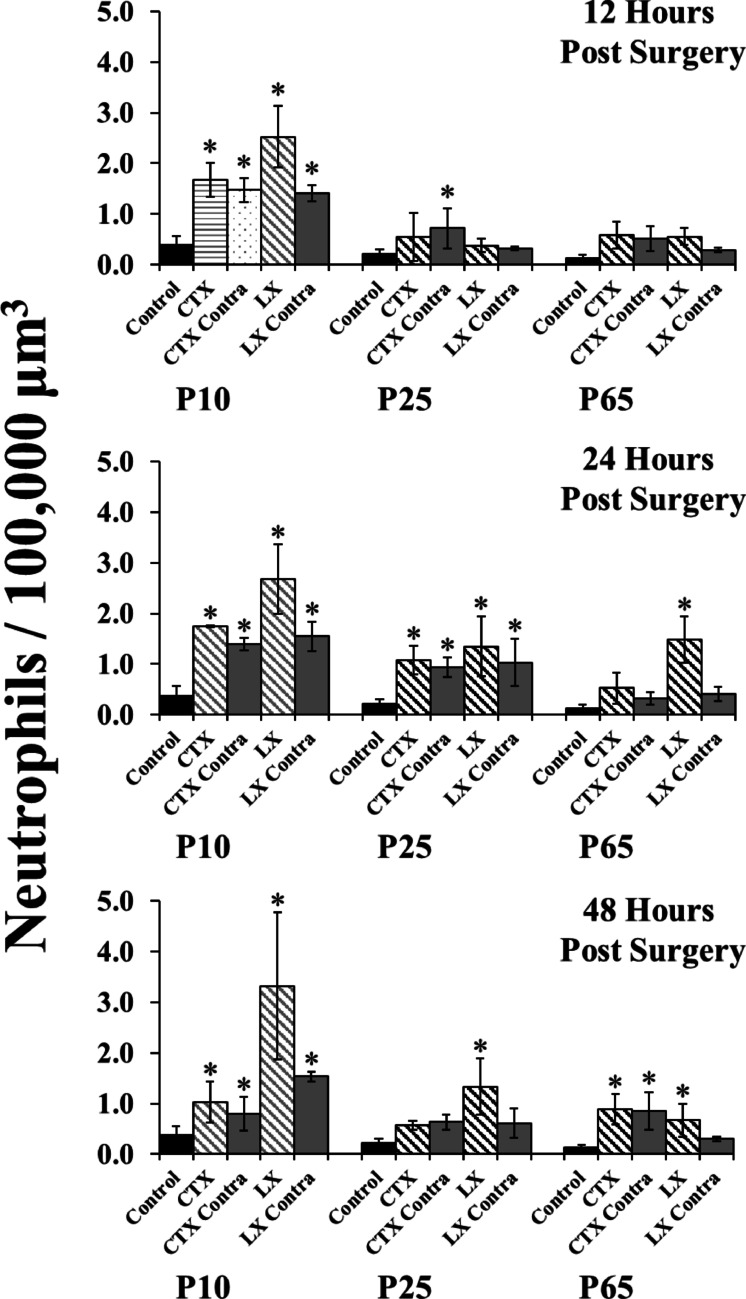
Neutrophil responses 12, 24, and 48 h following CTX or LX at key developmental ages. The largest and longest responses are observed when transections occur at P10. * indicates *p* < .05

A significant main effect of surgery type *F*(3, 87) 8.476, *p* < .001 and a significant interaction between surgical age and surgery type *F*(6, 87) = 2.518, *p* = .027, suggests that neutrophils respond differently based on surgery, an effect that further varied as a function of age (Fig. 3).

### Ipsilateral and contralateral neutrophil responses to neonatal surgeries are consistently elevated for 48 h

A robust neutrophil density increase was observed on the surgical side of the tongue at 12 h following P10 CTX *t*(7) = 6.614, *p* < .001 and LX *t*(7) = 10.987, *p* < .001, although the LX response was larger *t*(4) = 4.889, *p* = 008. The distribution of the neutrophils was not significantly impacted by either surgery (*p*s > 0.1), with the majority (~ 94%) being present in the muscle. Further, neutrophil density also increased on the contralateral side of the tongue at P10 for both CTX *t*(7) = 5.598, *p* = .< 0.001 and LX *t*(7), 3.938, *p* = .001, with the location not impacted by either surgery (*p*s > 0.1). The magnitude of the contralateral neutrophil density increases did not differ between CTX and LX (*p* > .1).

These effects remained consistent at the 24-hour mark: both CTX *t*(7) = 6.987, *p* < .001 and LX *t*(7) = 11.787, *p* < .001 had strong neutrophil responses that had not changed since the 12-hour mark (*p*s > 0.1), and the LX response continued to be larger (*p* = .005). Neutrophil location also remained consistent (*p*s > 0.1), with ~ 97% in the muscle. Elevated neutrophil density on the contralateral side also remained at 24 h following CTX *t*(7) = 5.156, *p* = .001 and LX *t*(7) = 5.986, *p* < .001, increases that did not differ based on surgery type *t*(4) = 0.928, *p* = .406, and had not increased since 12 h (*p*s > 0.1). Robust neutrophil responses remained elevated on both ipsilateral and contralateral sides through 48 h post injury (*p*s < 0.001).

### Neutrophil responses are delayed and acute in juveniles and adults

In contrast to P10 surgery, neither CTX nor LX induced neutrophil increases at 12 h post-injury in P25 or P65 rats (*p*s > 0.05). Yet by 24 h post-surgery, both P25 CTX and LX resulted in a significant increase in neutrophils on both the ipsilateral (*ps* < 0.01) and contralateral sides (*ps* < 0.01). These responses were smaller than those observed at P10 (*ps* < 0.001). Unlike at P10, the CTX and LX responses were comparable to each other (*ps* > 0.1). All P25 neutrophil responses returned to control levels by 48 h post surgery (*p*s > 0.05) with the exception of ipsilateral LX responses, which remained elevated *t*(7) = 5.728, *p* < .001.

For P65 animals, there was no neutrophil response to CTX for the first 24 h (*p*s > 0.05), although by 48 h neutrophils had increased on both sides of the tongue (*p*s < 0.01) to levels comparable to those at P10 (*ps* > 0.1). The LX response similarly was absent 12 h post injury (*p* > .05), yet was robust at the 24 h mark and remained high through 48 h (*p*s < 0.05). P65 LX responses remained lower than those observed at P10 (*ps* < 0.001).There was no response on the contralateral side (*p*s > 0.1).

## Discussion

In the current study we observed developmental variance in the neutrophil response to surgical transection of either the CT or LN, with a robust and prolonged neutrophil response observed neonatally that is delayed, acute, or absent later in life. This response is independent of baseline neutrophil quantities on the tongue, which are stable across development.

### Greater, earlier, and prolonged neutrophil response to neonatal transection corresponds to more severe consequences

While juvenile and adult CTX or LX results in transient consequences to taste buds and/or papillae (Sollars 2005; Omelian et al. 2016), neonatal CTX causes permanent loss of taste buds and structural deformities of papillae (Sollars et al. 2002; Sollars 2005), and neonatal LX results in permanently smaller taste buds than controls (Omelian et al. 2016). The permanent and more severe effects of neonatal nerve transections correspond with neutrophil responses that are earlier, larger, and last longer than those of juveniles and adults where full recovery occurs. Neutrophils are associated with injury repair (reviewed in Anitua et al. 2025; Torres-Ruiz et al. 2023), and prolonging the neutrophil response to adult CTX is deleterious to recovery (Steen et al. 2010), suggesting the potential that the longer neutrophil response to neonatal injuries may impact recovery. Further work is needed to see if relationship holds true earlier in development and for the lingual nerve. A robust neutrophil response was still present 48 h later in the current study, which suggests that there may also be a longer duration of response beyond the 48 h we examined. Our observations mirror what is seen following CTX in older adult and aging rats where the largest neutrophil increase is at 24 months of age (He et al. 2012). We propose an inverted parabolic neutrophil response curve to nerve transection that is large and prolonged neonatally, acute and lesser from P25-P65, then regresses back to a large response at P90, which continues to increase in magnitude to 24 months of age (He et al. 2012). Strikingly, this curve also parallels recovery; neonatal and aging rats have both a larger neutrophil increase and the poorer recovery outcomes than rats aged P25-P90 (He et al. 2012).

While the current study cannot rule out the possibility that the larger neutrophil response we observed is simply a consequence of more severe degeneration after neonatal injury, evidence from adult CTX paradigms (Steen et al. 2010) coupled with comparable neutrophil responses between CTX and LX despite the LN possessing over 5-times as many fibers as the CT (Farbman and Hellenkant 1978; Sollars et al. 2002) renders that possibility unlikely. Despite the CT and LN not innervating muscles directly, the neutrophil increase we observed remained primarily localized to the muscle, with no significant changes in neutrophil location in any condition. This may be explained in part by the disproportionate vascularization and substantial size differential of the muscle relative to the other locations we examined. When muscles or their associated motor nerves are injured, elevated neutrophil responses in the muscle can have lasting impacts on physiology (e.g. Bendszus et al. 2002; Fukushima et al. 2025), although it has yet to be established if this holds true for non-innervating nerves.

### Brain-derived neurotrophic factor and interferon gamma

Taste receptor cells are a source of brain derived neurotrophic factor (BDNF) on the tongue (Yee et al. 2005; Meng et al. 2017), which is required for proper gustatory nerve innervation across development (Mistretta el al. 1999; Krimm et al. 2010; Huang and Krimm 2010; Huang et al. 2015; Meng et al. 2015) and for re-innervation of taste receptor cells following CTX in adulthood (Meng et al. 2017). Neutrophil responses on the tongue can also be induced by systemic injection of lipopolysaccharide (LPS; Steen et al. 2010), and BDNF performs a protective function against LPS-induced neuroinflammation (Wei et al. 2015). Important to the results we observed, BDNF in tastebud progenitor cells decreases between P5 and P10, then remains relatively stable through to adulthood (Huang et al. 2015). In rats BDNF decreases with advanced age (Meng et al. 2017) consistent with decreases in recovery and increases in neutrophil response (He et al. 2012).

Neutrophils are a source of interferon gamma (IFN-γ; Ethuin et al. 2004; Spees et al. 2014; Gomez et al. 2015) and IFN-γ driven inflammation on the tongue inhibits the typically rapid taste receptor cell proliferation rates (10–21 day; Beidler and Smallman 1965; Hendricks et al. 2004; Hamamichi et al. 2006; Wang et al. 2007) and increases taste receptor cell death (Cohn et al. 2010). Treatments reducing inflammatory response in the 2 weeks following adult tongue injury have been shown to improve recovery outcomes (Ata et al. 2024). We therefore propose a potential mechanism for the parabolic neutrophil response curve to nerve transection: in ages with decreased BDNF (neonates and aged), the neutrophil response is largest, which in turn increases IFN-γ quantities and subsequently contributes to taste receptor cell death, decreased proliferation and, in some cases, lack of regeneration. Further research is needed to verify this possibility.

### Neutrophils on the tongue across development and contralateral effects of nerve transection

We report that the density and spatial distribution of neutrophils on the tongue remained stable across development (P10-P65). However, systemically, neutrophil density is approximately half that of adult levels during the first weeks of life when quantified in blood (Christensen and Rothstein 1985), or cell mass per body weight (Erdman et al. 1982). The current study reports comparable neutrophil increases on both ipsi- and contralateral sides of the tongue following P10 nerve transection, a phenomenon previously observed in P90 and 24 month old rats (He et al. 2012). Despite the unilateral nature of the CT, after adult CTX the contralateral side of the tongue undergoes structural (Li et al. 2015; Meng et al. 2017) and functional alterations (Wall and McCluskey 2008; Martin and Sollars 2015), the latter of which can also be induced by LPS (Steen et al. 2010) or aldosterone injections (Guagliardo et al. 2009). The innate immune response has been suggested as contributing to these changes and neutrophils are present in both sides of the tongue within 12 h of each type of injury (CTX or LPS; Steen et al. 2010). Indeed, following either unilateral CTX or LX, transient subnormal sodium responses on the contralateral, intact CT occur concurrently with elevated neutrophil quantities and are resolved when the immune response has subsided (Hendricks et al. 2002; Wall and McCluskey 2008).

The current investigation marks the first in-depth quantification of the peripheral immune response to injury of the developing gustatory and trigeminal systems. We demonstrated a greater neutrophil response to injury during early development relative to adulthood. The greater neonatal neutrophil response corresponds to previously demonstrated post-surgery effects that are more severe (Sollars 2005; Omelian et al. 2016), adding to previous evidence suggesting a role for the innate immune response in gustatory function and injury recovery (McCluskey 2004; Steen et al. 2010; Shi et al. 2012; Dong et al. 2023).

## Data Availability

The data that support the findings of this study are available from the corresponding author upon reasonable request.
